# Early language development in preterm children without neurological damage: a longitudinal study

**DOI:** 10.12688/f1000research.13314.1

**Published:** 2017-12-22

**Authors:** Micaela Capobianco, Luca Cerniglia

**Affiliations:** 1 Department of Psychology, Sapienza University of Rome, Rome, 00185, Italy; 2 International Telematic University Uninettuno , Rome, 00186, Italy

**Keywords:** Preterm children, biological risk, linguistic disorders, first verbal skills

## Abstract

Children born at a very low gestational age, even those without neurosensory damages, are at risk of linguistic disorders. This longitudinal study aimed at analyzing communicative and language abilities in preterm children during their second year of life, through a standardized questionnaire, with particular attention to the communicative and language abilities that predict the first verbal skills. Our results showed that preterm children are slower than full-terms in language acquisition particularly at earlier stages of development. The differences between the two groups of children was significant only at 16 and 18 months. Preterms use more simplistic linguistic categories for longer than full-terms, with regards to lexicon composition and syntactic complexity. This different pattern could involve more qualitative, rather than quantitative, aspects of developmental processes that characterize language acquisition in preterms and full-term children.

## Introduction

The study of early language development processes in children who were born at biological risk, such as preterm infants without neurosensory damages, is crucial. In fact, it has been shown that children born at very low gestational age are at risk of linguistic disorders in their first years of life ( Capobianco *et al*., 2010; Cimino *et al*., 2016). The present longitudinal study aimed at analyzing communication and language abilities in preterm children during their second year of life, through a standardized questionnaire for parents, the “Primo Vocabolario del Bambino” (Caselli *et al*., 2015) (PVB, Italian version of “MacArthur-Bates”, age 8–36 months) with particular attention to communication abilities that are predictors of the first verbal skills at 3 years ( Pizzuto & Capobianco, 2008; Capobianco *et al*., 2017).

## Method

### Participants

40 children participated in this study: 20 preterm children (7 females; 13 males) and 20 full-term children (7 females; 13 males). Inclusion criteria for preterm children were: a) No neurological damages at birth; b) APGAR between 7 and 10; c) Gestational age between 31 and 33 weeks; d) Weight appropriate for the gestational age. Preterm children had an average gestational age of 32 weeks (s.d.=2) and an average weight at birth 2200 gr. (s.d.=250). Inclusion criteria for full-term children were: a) No neurological damages at birth; b) Gestational age between 37 and 40 weeks; c) weight appropriate for the gestational age; d) no previous history of neurodevelopmental disorders. The average gestational age of full-terms was 39 weeks (s.d=3) and their weight at birth was 3500 gr. (s.d=300). All preterm and full-term children had a normal IQ (>85) evaluated by the Bayley Scales (II version) at 18 and 24 months of age. All participants were from an upper-middle-class family (calculated with an ad hoc questionnaire assessing the educational level and occupational status of parents). The preterm group and the full-term group were matched on age and sex.

Preterm children were recruited at the University Hospital of Rome, Policlinico Umberto I, (Puericulture Clinic), where all children at risk (including preterm children born with no neurological damage) undergo a protocol starting at birth for the monitoring of cognitive and language development every 3 months. Full-term children were selected from a larger longitudinal study on the spontaneous productions collection during the third year of age (Capobianco e Devescovi, 2008; Capobianco *et al*., 2017).

### Ethics and consent

All parents of children recruited for this study gave their written consent for participation in this research and for the publication of its results. The study was approved by the Ethical Committee of Sapienza, University of Rome (ID: 1/2007).

### Measures and procedure

The language ability of preterm and full-term children was examined at 16, 18, 20, 22 and 24 months of age through the Italian version of the MacArthur-Bates Communicative Development Inventories (MB-CDI questionnaire) (Caselli *et al*., 2015): at 16 months “Words and Gesture“ was used, Complete Form (validated for Italian toddlers of age from 8 to 24 months); at 18, 20, 22 and 24 months of age the other version labeled “Words and Phrases” was used (validated for Italian toddlers age from 18 to 36 months). Families of preterm children were asked to fill in the questionnaire during the clinical follow-up in Hospital. Families of full-term children were asked to fill in the questionnaire at their home every time the researcher went to the child’s home to collect the spontaneous production data. 

At 16 months the following indexes were derived by PVB questionnaire: word comprehension, word production (with lexicon composition: proportions of nouns, verbs and functors) gesture production. At 18, 20, 22 and 24 months the following measures were derived: word productions, lexicon composition (proportions of nouns, verbs and functors), number of phrases used and syntactic complexity with respect to the proportion of phrases classified as “with functional words” (e.g. “mommy car”) vs. “without functional words” (e.g. “the car of mommy”) used.

### Statistical analysis

Parametrical analysis T-test (t Student) and ANOVA (Analysis of Variance) were conducted to analyze the differences in language abilities between ages within each group (preterm ad full-term infants).

## Results

### Word comprehension and gesture production

At 16 months the difference between pre-terms and full-terms was significant for word comprehension [t _(38) _=2.19 _, _p<0.05] and gesture production [t _(38)_=3.79, p<0.01]. Examining lexicon composition (proportions) in comprehension at 16 months, we found that full-term children showed a higher percentage of Verbs (t (22)= -2.24, p= 0.03) and Functors (t (22)= -1.16, p= 0.07) than pre-terms. The differences between pre-terms and full-terms was significant for Verbs [t _(38) _= 2.16, p<0.05] and Nouns [t _(38)_=-2.08, p<0.05], but not for Functors [t _(38)_=0.931, n.s.] in comprehension.

### Word production

Results showed that preterms produced a lower number of words then full-term children at all assessment points (16, 18, 20, 22 and 24 months), although in the normative range of typical development (PVB). Moreover, the statistical analysis (ANOVA) showed a significant growth of lexicon production over time in both preterms and full-term children [F _4,35_=15.6, p<0.01]. The differences between preterms and full-terms was significant at 16 months [t _(38)_=4,05, p<0.01] and 18 months [t _(38)_=2.43, p<0.05], but not at subsequent assessment points (20, 22, 24 months). We also found that preterm children use less Verbs and Functors at all age. Nouns increased over time, and full-term children show a systematic increase of Verbs and Functors from 18 to 24 months, and a decrease of Nouns over time [20 months: t _(38)_=2.25, p<0.05; 22 months: t _(38)_=2.29, p<0.05].

### Phrases production

**Figure 1.**
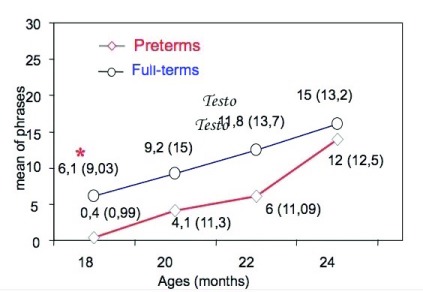
Number of phrases produced by preterms and full-terms from 18 to 24 months.

**Figure 2.**
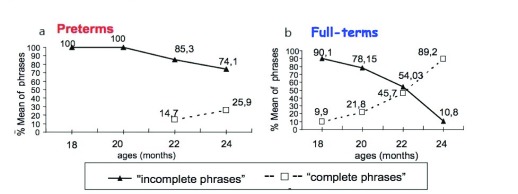
( **a**) Phrases “incomplete” and “complete” in preterms from 18 to 24 months ( **b**) phrases “incomplete” and “complete” in full-terms from 18 to 24 months.

Figure 1 shows the number of phrases produced by preterm children and full-terms at 18, 20, 22 and 24 months. Figure 2a and the Figure 2b show respectively the number of phrases with and without functional words produced by preterm children and full-terms at 18, 20, 22 and 24 months. Preterm children used a lower number of phrases than full-terms at all ages. Statistical analysis (ANOVA), however, showed a significant growth of phrase production over time in both preterms and full-terms [F _3.36_=12.12, p<0.01]. The differences were significant only at 18 months [t _(38)_=2.80, p<0.05] but not at subsequent assessment points [F _3.36_=12.12, p<0.01]. At 24 months (last observation) we found that preterm and full-term children produced a similar number of phrases [preterm=13.9 phrases; full-term=13.2 phrases]. We found significant differences in the use of phrases classified as “with and without functional words” ( Figure 2a and Figure 2b) [F( _3.36)_=6.29, p<0.01]. However, the data showed that preterms used more phrases classified as “without functional words” at all age and phrases classified as “with functional words” were produced only at 22 months (14.7 %), increasing at 24 months (25.9 %). In contrast, full-terms used the phrases classified as “with functional words” at the first observation (18 months) and the frequency of this category of phrases increased between 20 and 24 months. The use of phrases classified as “without functional words” decreased in full-terms over time. At 24 months full-terms produced significantly more phrases classified as “with functional words” (89.2 %) than phrases classified as “without functional words” (10.8 %).

## Discussions

Our data showed that preterms were slower than full-term children in language acquisition especially at earlier stages of development. Even if preterms had a reduced vocabulary in general, the differences between the two groups of children were significant only at 16 and 18 months. Moreover, preterm children tended to a naturally recover primary acquisitions during the second year of life. The differences were present in the qualitative aspects of language abilities, such as the lexicon and verbal combinations at 24 months. We observed that preterms used more simplistic linguistic categories longer than full-terms, more simplistic linguistic categories referring to lexicon composition (nouns) and syntactic complexity (phrases without functional words). These different patterns could involve more qualitative rather than quantitative aspects of developmental processes that characterize language acquisition in preterms and full-term children. This data have several clinical and research implications. First, it can be useful for the early prevention of language disorders in preterm children, through the screening of the specific elements of lexicon composition and of phrase complexity. Second, they confirmed the importance of longitudinal studies in this field and the usefulness of chronological age in assessing the early language development of preterm children comparing them with full-term offspring, to observe the early recovery and the qualitative differences in language production.

## Data availability

The data referenced by this article are under copyright with the following copyright statement: Copyright: © 2017 Capobianco M and Cerniglia L

Data associated with the article are available under the terms of the Creative Commons Attribution Licence, which permits unrestricted use, distribution, and reproduction in any medium, provided the original data is properly cited. 

All raw data is accessible at: http://dx.doi.org/10.17632/zgvk4hb8hb.2 ( Cerniglia, 2017)

Data are available under the terms of the Creative Commons Attribution 4.0 International license (CC-BY 4.0).
