# Supplementary material for: Early language development in preterm children without neurological damage: a longitudinal study
Source: F1000Res. 2017 Dec 22;6:2169. [Version 1] doi: 10.12688/f1000research.13314.1 (PMC5791006; doi:10.12688/f1000research.13314.1)
Supplement: Peer review history

# Peer review: Referee response for version 1

**DOI:** 10.5256/f1000research.14448.r29991

**Published:** 2018-01-29

**Authors:** Allison B. Ellawadi

**Affiliations:** 1 Department of Speech and Hearing Science, Ohio State University, Columbus, OH, USA

Thank you very much for the opportunity to review this interesting and well written report. My comments for the statistical analysis and discussion are listed below.

 Statistical Analysis:

- Word comprehension and gesture productions – a Multivariate Analysis of Variance using group as the independent variable and gesture and word comprehension as the dependent variables. A follow-up analysis using the different lexical categories would then be appropriate.
- In the Word Production Analyses – the authors note that “We also found that preterm children use less Verbs and Functors at all age. Nouns increased over time, and full-term children show a systematic increase of Verbs and Functors from 18 to 24 months, and a decrease of Nouns over time [20 months: t(38)=2.25, p<0.05; 22 months: t(38)=2.29, p<0.05].” This is a bit confusing. Does this indicate that the preterm infants lost some of the nouns that they were producing (e.g., parents reported that they produced 25 nouns at 20 months but then only 10 nouns at 24 months?). The information in this section appears contradictory with regard to the noun data reported.

 

 Discussion:

- It seems to point to the children having initially slower language acquisition but then they must demonstrate a faster rate of growth at some point (or the full term infants demonstrate a slowed rate of growth) since there is not a difference in words and phrases by 24 and the only different in vocab was at 16 and 18 months. While the authors state this later on in the first paragraph, this is not immediately clear. I would recommend starting with something that states that although there are initial delays the preterm infants appear to catch up.
- The authors should put in a couple of more sentences to discuss what is meant by “qualitative aspects” of language abilities and why it mattes that preterms seem to have differences in these aspects of language later in in development.

I have read this submission. I believe that I have an appropriate level of expertise to confirm that it is of an acceptable scientific standard.

# Peer review: Referee response for version 1

**DOI:** 10.5256/f1000research.14448.r29655

**Published:** 2018-01-15

**Authors:** Laura Serra

**Affiliations:** 1 Neuroimaging Laboratory, Santa Lucia Foundation, Scientific Institutes of Hospitalization and Care (IRCCS), Rome, Italy

I have reviewed the Research Note “Early language development in preterm children without neurological damage: a longitudinal study”. I think that the paper is well written and offers a relevant contribution to studies on the early communicative and language development in preterm children without neurological damage.

 I accept the work with minor revisions. In particular:

1. I think it's necessary to add in the Reference list a more recent article on the preterms early language developmental.
2. There is a mismatch for the citation of Questionnaire “PVB” (1995 or 2017?) between Reference list and text.
3. I suggest adding a very brief passage about the reasons why the authors chose those measures over others.

I have read this submission. I believe that I have an appropriate level of expertise to confirm that it is of an acceptable scientific standard.
